# Burnout syndrome and workplace violence among nursing staff: a cross-sectional study

**DOI:** 10.1590/1516-3180.2021.0068.R1.31052021

**Published:** 2021-12-17

**Authors:** Sirlene Aparecida Scarpin Tsukamoto, Maria José Quina Galdino, Maynara Fernanda Carvalho Barreto, Júlia Trevisan Martins

**Affiliations:** I MSc. Nurse, Hospital Evangélico de Londrina, Londrina (PR), Brazil; and Member of the Núcleo de Estudos da Saúde do Trabalhador, Universidade Estadual de Londrina (NUESTUEL), Londrina (PR), Brazil.; II PhD. Nurse and Adjunct Professor, Department of Nursing, Universidade Estadual do Norte do Paraná (UENP), Bandeirantes (PR), Brazil; and Coordinator, Study Group on Teaching, Health and Work, Universidade Estadual do Norte do Paraná (UENP), Bandeirantes (PR), Brazil.; III PhD. Nurse and Professor, Department of Nursing, Universidade Estadual do Norte do Paraná (UENP), Bandeirantes (PR), Brazil; and Member of the Study Group on Teaching, Health and Work, Universidade Estadual do Norte do Paraná (UENP), Bandeirantes (PR), Brazil.; IV PhD. Nurse and Associate Professor, Department of Nursing, Universidade Estadual de Londrina (UEL), Londrina (PR), Brazil; and Coordinator, Study Group on Occupational Health, Universidade Estadual de Londrina (UEL), Londrina (PR), Brazil.

**Keywords:** Mental health, Violence, Workplace violence, Exposure to violence, Physical abuse, Mental hygiene, Violence exposure, Physical violence, Physical health-related injuries, Psychological health-related injuries, Workplace exhaustion.

## Abstract

**BACKGROUND:**

Among healthcare professionals, nursing workers are the most prone to becoming victims of workplace violence and present the highest burnout levels.

**OBJECTIVES:**

To investigate the association between burnout syndrome and workplace violence among nursing workers.

**DESIGN AND SETTING:**

Cross-sectional study carried out at a teaching hospital in southern Brazil.

**METHODS:**

This study involved 242 nursing workers. We collected data over a six-month period using a sociodemographic and occupational survey, the Survey Questionnaire Workplace Violence in the Health Sector and the Maslach Burnout Inventory - General Survey. For occupational violence, we selected the Survey Questionnaire Workplace Violence in the Health Sector. Burnout syndrome was evaluated using the Maslach Burnout Inventory - General Survey. The data were analyzed in the Statistical Package for the Social Sciences (SPSS). Categorical variables were described as absolute and relative frequencies and numerical variables in terms of central trend and dispersion measurements. For data analysis, we applied descriptive statistics and multiple logistic regression.

**RESULTS:**

The multiple models indicated that the workers who had experienced verbal abuse, physical violence and concern about workplace violence over the past 12 months had significantly higher chances of presenting high emotional exhaustion (P < 0.05) and depersonalization (P < 0.05) and low professional accomplishment (P < 0.05).

**CONCLUSION:**

Occurrence of violence significantly increased the chances of great emotional exhaustion and depersonalization and low professional achievement, within burnout syndrome. Therefore, workplace violence prevention strategies need to be put in place to provide workers with a safe workplace in which to conduct their activities.

## INTRODUCTION

Workplace violence has become a worldwide public health problem.^1^^,^^2^ All workers are exposed to violence, but healthcare professionals are at potential risk of experiencing physical or verbal abuse while performing their work activities.^3^ Workplace violence is defined as any action, incident or behavior based on a voluntary procedure of the aggressor, as a result of which a professional is assaulted, threatened or suffers any damage or injury during the performance of his or her work, or as a direct result of the work.^4^

The harmful effects of violence on healthcare workers have been acknowledged in both developed and developing countries,^5^ and have become a growing phenomenon.^6^ In 2013, in the United States, there were around 24,000 cases of workplace violence per year, among which more than 70% occurred within healthcare services. This finding shows that workplace violence is almost four times more likely to be experienced by healthcare workers than by workers in other sectors.^7^ Physical violence and sexual harassment are more prevalent in Anglo-Saxon countries, and verbal abuse in the Middle East.^1^^,^^8^

Workplace violence within healthcare comes from patients, caregivers, physicians and, co-workers and depends on the social characteristics of the subjects and the hospital environment.^9^ Thus, nursing teams, which interact more closely with patients and caregivers and are present in hospitals 24 hours a day, end up being one of the main victims of violence.^10^ In addition, nursing workers are the target of physical, verbal and psychological violent behavior, sexual harassment and lack of support and trust coming from colleagues, superiors and managers.^11^^,^^12^

When experiencing workplace violence, nursing professionals are predisposed to physical and psychological health-related injuries, which may affect the workers’ ability to perform their daily activities.^13^ Violence is related to occurrence of workplace accidents and absenteeism and negatively affects worker satisfaction and recognition.^14^

In addition, violence can affect the entire workforce and consequently impair the quality of care provided to patients and their families. The negative consequences of workplace violence for workers’ health have been displayed through symptoms of stress, low self-esteem and discouragement among the victims. These are symptoms that trigger burnout syndrome.^15^

Burnout syndrome is manifested as a prolonged response to chronic interpersonal stressors. This process consists of three dimensions: emotional exhaustion, depersonalization and low professional accomplishment. The meaning of this three-dimensional model is that it clearly places the experience of individual stress within a social context and involves conceptions of oneself and others. As a consequence of the syndrome, work loses its meaning, thereby generating demotivation, negative attitudes and distancing, which cause losses within the health-work process.^16^

Investigating workplace violence and its association with burnout syndrome among nursing professionals is relevant due to the lack of studies on this subject in Latin America.^17^ Thus, the results will enable further knowledge about workplace violence and its relationship with burnout syndrome and can contribute to development of improved violence prevention strategies and decreased workplace exhaustion, which will improve the quality of life in the workplace.

## OBJECTIVE

The objective of this study was to investigate the association between burnout syndrome and workplace violence among nursing workers.

## METHODS

This was a cross-sectional study carried out at a teaching hospital in southern Brazil that offers 313 beds through the Brazilian National Health System (Sistema Único de Saúde, SUS) and provides medium and high-complexity healthcare.

During the study period, the population was composed of 680 nursing workers. To calculate the sample size, a formula for finite populations was used, in which the outcome was taken to be 50% prevalence and a 95% confidence interval was assumed. Through this, the minimum number of workers to be included in the study was 242.

The inclusion criterion for this study was that the subjects needed to have been in their current job for at least one year, in order to avoid bias due to occupational adaptation. Workers who were on holiday or other leave during the data collection period were also excluded.

Data were collected from January to June 2018, through a sociodemographic and occupational survey, which evaluated workplace violence and burnout syndrome. The primary author clarified the purpose of the research and provided the workers with the instrument at the workplaces during their working hours. They were instructed to place the completed questionnaire in a sealed box that was available in all the places where data were collected.

This characterization questionnaire asked for the following sociodemographic data: age (in years), sex (female or male), marital status (single, married, divorced or cohabiting) and schooling (in number of years); and the following occupational data: professional category (nurse or auxiliary nurse/nursing technician), length of service at the institution (in years), weekly workload (in hours, dichotomized as ≤ 36 hours or ≥ 37 hours) and work shift (day or night).

To assess occupational violence, we selected the Survey Questionnaire Workplace Violence in the Health Sector, which was developed by the World Health Organization, International Labour and Public Service Organization and International Council of Nursing.^18^ From this instrument, we used the questions about experiences of physical violence, verbal abuse, sexual harassment and other types of violence in the workplace over the previous 12 months. This questionnaire is not a construct that generates a score, which thus allowing analysis on different types of violence separately.

Burnout syndrome was evaluated using the Maslach Burnout Inventory - General Survey (MBI-GS). This is a scale elaborated by Maslach and Jackson, and the Brazilian version was translated by Tamayo in 1997 and validated by Schuster et al. in 2015. It presents good reliability, with Cronbach’s alpha between 0.82 and 0.84.^19^ It is a self-report questionnaire consisting of 16 assertions, accompanied by seven-point Likert-type responses (0-6). The dimension of emotional exhaustion is evaluated by means of six items, depersonalization is evaluated through four items, and low professional accomplishment is evaluated through six items.

The data were analyzed in the Statistical Package for the Social Sciences (SPSS), version 20.0 (IBM, Chicago, Illinois, United States). The categorical variables were described in terms of absolute and relative frequencies and the numerical variables in terms of central trend and dispersion measurements.

The outcomes were the dimensions of the burnout syndrome: emotional exhaustion, depersonalization and professional accomplishment. These were classified as high or low, based on the median.^16^ Associations between these dimensions and workplace violence were initially examined using univariate logistic regression and then unadjusted and adjusted multiple logistic regression. The multiple models were adjusted according to sex, age and work shift, which have been indicated in the literature to be control variables.^20^^,^^21^ The goodness of fit of the models thus elaborated was verified through the Hosmer-Lemeshow test, in which the higher the alpha value was, the better the fit also was. P-values < 0.05 were considered statistically significant.

Institutional review board approval was sought and obtained, in accordance with opinion report no. 2.386.855. Licenses to use the MBI-GS were purchased from Mind Garden, which manages the inventory’s copyright.

This study was approved by our institution’s ethics committee for research involving human subjects in November 2017. Its Brazilian certificate of presentation for ethics assessment (CAAE) number for public consultation is 78866017.1.0000.5231.

## RESULTS

In total, 242 nursing workers aged between 20 and 68 years, and with an average age of 43 years, participated in this study. The majority of them were female (74.4%), worked as technicians and auxiliary nurses (71.9%) and developed their work activities during the day (60.7%) (Table 1).

**Table 1.** Characteristics of study participants

Variables (n = 242)	Mean (standard deviation)	n (%)
Age (in years)	43.4 (9.4)	
Sex		
Male		62 (25.6)
Female		180 (74.4)
Marital status		
Single		117 (48.3)
Married		125 (51.7)
Education (in years of schooling)	15.4 (3.3)	
Professional category		
Nurse		68 (28.1)
Auxiliary nurse/nursing technician		174 (71.9)
Length of service at the institution (in years)	12.9 (9.7)	
Weekly workload in hours		
≤ 36 hours		122 (50.4)
≥ 37 hours		120 (49.6)
Work shift		
Day		147 (60.7)
Night		95 (39.3)

Physical and verbal violence, sexual harassment and concern about workplace violence were significantly associated with all dimensions of burnout syndrome, except between sexual harassment and depersonalization. High emotional exhaustion was associated with physical violence (P < 0.001), verbal abuse (P < 0.001), sexual harassment (P = 0.002) and concern about workplace violence (P = 0.005). High depersonalization was associated with physical violence (P < 0.001), verbal abuse (P < 0.001) and concern about workplace violence (P = 0.002). Low professional accomplishment was associated with physical violence (P = 0.001), verbal abuse (P = 0.001), sexual harassment (P = 0.031) and concern about workplace violence (P = 0.002) (Table 2).

**Table 2.** Association between workplace violence and the dimensions of burnout syndrome among nursing workers

Variables (n = 242)	Emotional exhaustion		Depersonalization		Professional accomplishment	
	high	P-value	high	P-value	low	P-value
	n (%)		n (%)		n (%)	
Physical violence						
No	75 (38.9)	< 0.001	84 (43.5)	< 0.001	97 (50.3)	0.001
Yes	37 (75.5)		37 (75.5)		38 (77.6)	
Verbal abuse						
No	26 (26.3)	< 0.001	35 (35.4)	< 0.001	42 (42.4)	0.001
Yes	86 (60.1)		86 (60.1)		93 (65.0)	
Sexual harassment						
No	89 (42.2)	0.002	101 (47.9)	0.088	112 (53.1)	0.031
Yes	23 (74.2)		20 (64.5)		23 (74.2)	
Concern about workplace violence						
No	53 (38.4)	0.005	57 (41.3)	0.002	65 (47.1)	0.002
Yes	59 (56.7)		64 (61.5)		70 (67.3)	

The multiple models indicated that the workers who had experienced verbal abuse, physical violence and concerns about workplace violence in the past 12 months presented significantly higher chances (P < 0.034) of high emotional exhaustion, high depersonalization and low professional accomplishment. Conversely, sexual harassment was not significantly associated (P > 0.05) with the dimensions of burnout syndrome when the other types of violence were included in the model (Table 3).

**Table 3.** Influence of workplace violence on the dimensions of burnout syndrome among nursing workers

Multiple models (n = 242)	Odds ratio - unadjusted (95% confidence interval)	P-value	Odds ratio - adjusted (95% confidence interval)^*†^	P-value
Emotional exhaustion				
Verbal abuse	3.419 (1.876-6.231)	< 0.001	3.520 (1.916-6.468)	< 0.001
Physical violence	3.716 (1.748-7.900)	0.001	3.653 (1.697-7.861)	0.001
Sexual harassment	2.383 (0.964-5.891)	0.060	2.305 (0.898-5.914)	0.082
Concern about violence at work	1.885 (1.062-3.348)	0.030	1.874 (1.049-3.347)	0.034
Depersonalization				
Verbal abuse	2.378 (1.342-4.211)	0.003	2.479 (1.387-4.432)	0.002
Physical violence	3.308 (1.577-6.936)	0.002	3.158 (1.490-6.691)	0.003
Sexual harassment	1.192 (0.512-2.774)	0.684	1.314 (0.544-3.174)	0.543
Concern about violence at work	2.213 (1.270-3.857)	0.005	2.253 (1.285-3.951)	0.005
Low professional accomplishment				
Verbal abuse	2.010 (1.146-3.525)	0.010	2.684 (1.250-5.763)	0.011
Physical violence	2.701 (1.267-5.757)	0.015	2.031 (1.153-3.578)	0.014
Sexual harassment	1.615 (0.659-3.957)	0.294	1.533 (0.607-3.873)	0.366
Concern about violence at work	2.115 (1.214-3.684)	0.008	2.105 (1.205-3.675)	0.009

^*^Adjustment variables: sex, age and work shift; ^†^Hosmer-Lemeshow test on adjusted models: P = 0.712, P = 0.304 and P = 0.980, respectively.

## DISCUSSION

The predominance of women in this study was in accordance with data from research carried out in other countries.^22^^,^^23^ These data are culturally confirmed in nursing, as women have a series of experiences aimed at the construction of female abilities and skills, one of which is taking care of people;^24^ and the most characteristic and defining attribute of nursing is care.

In relation to female gender and burnout, women may have higher levels of stress and depersonalization in the work environment due to their personal characteristics and functions outside of work, such as domestic work.^25^ Regarding the professional category, most participants in the present study were nursing technicians or auxiliary nurses, which is common in the Brazil reality, as these categories represent 80% of nursing professionals.^26^ In other countries, however, most of them are nurses.^27^^,^^28^^,^^29^

Currently, many healthcare professionals worldwide report having experienced workplace violence^27^^,^^30^ and are consequently presenting psychological illness due to these experiences, such as the burnout syndrome.^31^ These data were verified in this study, in which professionals’ experiences of physical and verbal violence were associated with burnout syndrome.

In the United Kingdom, burnout and occupational violence are significant problems, as it has been estimated that 42% of nurses in that country are considered burned out.^32^ More than 55,000 physical assaults against National Health Service (NHS) teams have been reported, which has led many hospitals to conduct risk assessment actions for service users, educate staff and use safety teams,^33^ which are considered to be effective preventive measures.^34^

Becoming a target of workplace violence has a negative effect on workers’ wellbeing^35^ and causes psychological and physical harm, as well as extreme insecurity. These factors contribute to negative sentiments about work.^36^ The ensuing feeling of low personal valuation makes workers experience high levels of stress,^37^ which affect their interpersonal relationships, quality of life and ability to perform daily activities”.^13^ This also harms the victims in other regards: spiritually, morally and socially.^38^

In a study among nurses at 11 public hospitals in Spain, greater exposure to workplace violence was associated with greater emotional exhaustion and depersonalization, as well as with lower levels of psychological wellbeing.^39^ Studies conducted in Brazil and Turkey identified that healthcare workers who experienced or were exposed to workplace violence showed high levels of emotional exhaustion and depersonalization and low levels of professional accomplishment, and that workers who were victims of various forms of violence experienced this harm even more strongly.^14^^,^^40^ Similar findings were obtained among nurses in Lebanon and Palestine.^41^^,^^42^ These results are consistent with the findings obtained from the present study, in which nursing workers who had experienced physical violence and verbal abuse in the previous 12 months showed higher chances of presenting high exhaustion, depersonalization and low professional accomplishment.

Verbal aggression has been found to be the most prevalent among the types of violence that nursing professionals experience. It entails emotional exhaustion that activates a cycle of losses that can lead to dissipation of these individuals’ mental and physical resources. Moreover, as a result of perceiving this violence as a threat, workers may adopt an attitude of disengagement, such as depersonalization.^43^

Nursing professionals’ exposure to violence tends to contribute to development of a negative and demotivated response to work, which negatively affects their mental health.^44^ This exposure gives rise to feelings of hopelessness, disappointment, fear and anxiety.^42^

A study conducted in 23 hospitals in Guangdong province, in China, among 1,502 nurses, showed that violence in the workplace was directly associated with higher incidence of burnout, lower job satisfaction, lower patient safety and adverse events. Burnout was directly associated with lower patient safety and more adverse events.^45^

Studies in Turkey and Switzerland have shown that nurses who experienced verbal and physical abuse felt a strong desire to leave the profession. The fear that workplace violence will recur makes many of its victims more disposed to breaking their institutional bonds.^9^^,^^46^

In the present study, sexual harassment was only a statistically significant correlate when analyzed in isolation from other types of violence. Nonetheless, this link is a cause for concern, given that it did occur among the workers in this analysis. A previous study showed weak correlations between sexual harassment and the dimensions of burnout syndrome.^47^

It is a fact that sexual harassment often remains unreported because of its stigmatization.^48^ The traditional nature of hospital environment hierarchies can lead the victims of sexual harassment to ignore it due to shame and the assumption that reporting it will make no difference, especially if the perpetrator is a physician or a boss.^49^ Moreover, in Brazil, the media still attaches a negative stereotype to nursing by presenting the nurse’s body as a sexual object. Thus, professional organizations need to mobilize to link the image of nursing to excellent human care delivery and management.

Furthermore, in the present study, concern about workplace violence was associated with high emotional exhaustion, high depersonalization, and low professional accomplishment. According to research conducted in another Brazilian state, only 17.8% of the interviewees said that they were not worried about violence.^50^

Nursing professionals who have been victims of workplace violence, as well as those who believe that there is a possibility of violence, are worried. Feelings of distress can emerge. Consequently, physical or mental illness may occur.^51^

Thus, it is imperative to ensure the safety of healthcare workers in order to prevent health problems, sick leave and dropout from the job, along with social isolation and the intention to quit this work.^14^^,^^52^

There were limitation to this study that related to the self-assessment method used. This may have led to responses that were tailored in accordance with socially acceptable standards. This study was conducted at a public teaching hospital that is rated outstanding because of the high level of medical and nursing care that it makes available in this state in Brazil. Nevertheless, the sample may not have been representative given that convenience sampling was used, which prevents generalization of the results. Additional research is recommended at philanthropic and private hospitals, also focusing on the use of prevention strategies.

Despite these limitations, this study has contributed to advancement of scientific knowledge through revealing information about the different forms of violence that nursing professionals experienced in their workplace and how this problem was associated with burnout syndrome. Thus, it is essential that joint actions in this field should be planned and implemented by nursing workers and managers, in order to promote health and prevent injuries. Such measures should seek to enhance a safe working environment for all parties, and organizational support for these measures should act as a mediator between workplace violence, job satisfaction and burnout levels.^53^^,^^54^

## CONCLUSION

Workers who experienced verbal abuse, physical violence and concern about workplace violence in the previous 12 months showed significantly higher chances of high emotional exhaustion, depersonalization and low professional accomplishment.

Workplace violence prevention strategies need to be put in place, including workplace monitoring, so as to stimulate the reporting of violence, promote victim support networks and implement specific pre and post-intervention protocols for the different types of workplace violence, in order provide nursing workers with a safe workplace for development of their activities.
